# Exploring the Impact of Book Club Participation on Clinicians’ Empathy and Reflection on Empathetic Practice: A Wake-Up Call

**DOI:** 10.7759/cureus.39656

**Published:** 2023-05-29

**Authors:** Fei Chen, Kelly Powers, Amy M Goetzinger, Seema Patidar, Amy J Moulthrop, Christopher Pamacheche, Susan Martinelli

**Affiliations:** 1 Department of Anesthesiology, University of North Carolina School of Medicine, Chapel Hill, USA; 2 School of Nursing, University of North Carolina at Charlotte, Charlotte, USA; 3 Department of Anesthesiology, University of California, San Francisco, USA

**Keywords:** book club, medical education, humanistic medicine, anesthesiology, empathy

## Abstract

Introduction: Empathy is associated with desirable outcomes in healthcare, including improved patient-clinician rapport, fewer patient complications, and reduced clinician burnout. Despite these benefits, research suggests empathy declines during professional training. This study aimed to explore the impact of book club participation on clinicians’ and trainees’ empathy and perspectives on empathetic patient care.

Methods: In this mixed-methods study, anesthesiology clinicians and trainees were invited to respond to a baseline online empathy survey followed by an invitation to read a book and to participate in one of four facilitated book club sessions. Post-intervention empathy was measured. The primary outcome of the quantitative analysis was a change in empathy scores as measured by the Toronto Empathy Questionnaire. A thematic analysis of book club sessions and open-ended comments in the post-intervention survey was conducted.

Results: Participants included 74 responders to the baseline survey and 73 responders to the post-intervention survey. Empathy score change in the book club participants was not statistically significant from those who did not participate in any book club sessions (F_(2, 39)_ = 0.42, p=0.66). Thematic analysis of the book club sessions revealed four themes that highlight how the book club enhanced empathy awareness among trainees and clinicians: 1) a wake-up call, 2) deciding whether to take action, 3) learning and nurturing empathy, and 4) changing the culture.

Conclusion: There were no significant changes in empathy scores associated with book club participation. Thematic analysis highlighted barriers toward empathetic patient care, areas for improvement, and voiced intentions to practice with heightened empathy. Book clubs may be a viable venue to nurture a culture of increased self-awareness and motivation to counteract loss of empathy, but just one experience may not be sufficient.

## Introduction

Empathy is an essential constituent of humanistic healthcare. It is a multi-dimensional construct with affective, behavioral, and cognitive components. In clinical practice, empathy is defined as the ability to understand the patient’s situation, perspective, and feelings; communicate that understanding and check its accuracy; and act on that understanding with the patient in a helpful way [1].

Empathy has been associated with desirable patient care outcomes such as improved communications, stronger patient-practitioner rapport, enhanced diagnostic accuracy, reduced psychological stress, and lower complication rates [2-7]. Empathy has also been shown to reduce clinician burnout and malpractice risk due to improved physician communication [3]. Furthermore, empathy has been identified as a significant factor contributing to cultural competence [8].

Despite the demonstrated benefits of empathy for patients and clinicians, a decline in empathy occurs starting in the middle of medical training, particularly in the US [7]. Without intervention, such trends continue through residency and beyond [3,4,9,10]. Such a decline in empathy may be related to stress and burnout among medical students and residents when they get increased patient contact, long work hours, and sleep deprivation during clinical training [4,9].

Clinical empathy can be taught and improved [1,11,12]. Systematic reviews suggest that empathy-enhancing interventions can effectively cultivate and sustain empathy in trainees and clinicians [2,13]. However, the effect of training seemed to diminish over time, and most interventions focused on nursing and medical students. The majority of studied interventions were one-time implementations of empathy education through didactics, role play, experiential teaching, patient-preceptor feedback, and self-reflection to improve clinical communication skills and behaviors [2,13]. Yet, remediating the loss of empathy requires more than instruction in communication skills, it entails developing a culture that nurtures self-awareness of empathy and humanities in practice [3]. 

This study aimed to explore the effect of a facilitated book club intervention on anesthesiology clinicians’ and trainees’ empathy and their perspectives on empathetic care. We hypothesized that participating in the book club would improve clinicians’ and trainees’ empathy and encourage practice change toward empathetic patient care. To understand the underlying processes that produce and nurture empathic practice, we summarized the themes that emerged during book club sessions.

Part of this article was previously presented as a poster at the 2021 Virtual Well-Being Scholarship Expo at the University of North Carolina in December 2021.

## Materials and methods

This mixed-methods study was deemed exempt by the Office of Human Research Ethics of the University of North Carolina at Chapel Hill (Study #: 19‐3001). Participants provided informed consent to participate in the study by responding to the survey or book club session invitations. At the beginning of each book club session, the participants were verbally informed again as a group of the purpose of the study and that the study materials will be anonymized.

Participants and procedures

A convenience sample of clinicians and trainees in the Department of Anesthesiology at one academic medical center (University of North Carolina School of Medicine, Chapel Hill) in the southeast US was recruited. Those who consented to participate were invited to 1) take a baseline survey, 2) read the selected book, and 3) take a post-intervention survey. During the same consent process, the participants had the option to sign up for one of four facilitated book club sessions that were scheduled to occur after participants read the book but before completing the post-intervention survey, understanding that we would use the book club session transcripts for qualitative analysis. To engage all participants during the book club sessions, we set a cap of 10 participants per book club session and the actual number of participants per session varied due to their availability for the four pre-determined dates/times. Thus, only a subset of the participants who completed the surveys and/or read the book participated in the book club sessions. The book "In Shock" was selected because it was written by a physician who became a patient in the intensive care unit (ICU) and highlights lapses in empathy that can be seen in healthcare [14]. The perspective of a physician as a patient is rather unique and potentially a relatable narrative for clinicians. In addition, much of the book takes place in ICU and operative environments, making it especially relevant to technology-oriented subspecialties.

All clinicians (100 attendings, 60 residents, 88 certified registered nurse anesthetists (CRNAs), and 11 advanced care providers from the cardiothoracic ICU) in the anesthesiology department were invited via email, to take a brief online survey assessing their empathy and demographics. Participants were then invited via email to read the book In Shock and participate in one of four facilitated book clubs. The department distributed copies of the book; those who signed up for the book club received a complimentary copy. Clinical health psychologists (AMG, SP) facilitated the book club sessions, as they are experienced in formal group interventions. Each session lasted between 50 and 80 minutes and consisted of four to nine clinicians with varying roles and levels of experience. The discussions were guided by semi-structured questions to facilitate reflection on the book and practice experiences, maintain consistency between groups, and elicit views about the use of book clubs in medical education. Book club discussions were recorded using a local camera for the first in-person session and then via Zoom as sessions moved online due to the pandemic. The recordings were transcribed for qualitative analysis. The brief post-intervention survey was emailed to all clinicians in the department following these sessions.

Measures

Anonymous pre- and post-book club surveys were administered online via Qualtrics in the format of a general link. No IP address or any identifiable information was collected. Manufactured project identifier questions were added to the surveys to allow for matching baseline and post-intervention survey responses. The baseline survey contained the 16-item Toronto Empathy Questionnaire (TEQ) [15] and demographic questions including amount of clinical experience and whether they had previously read the book. The post-intervention survey included the TEQ questionnaire and project identifier question, as well as questions about the book club experience, views about the book, and change in practice questions. 

Data analysis

The TEQ empathy scores collected from both surveys were calculated and summarized. To understand the status quo of empathy score distribution in this sample, Kruskal-Wallis tests were used to compare baseline (pre-survey) empathy scores between groups of varying clinician roles (i.e., attending, CRNA, resident, other) and experience (i.e., less than five years, 5-10 years, and over 10 years). This analysis included all responses to the baseline survey regardless of whether the participants completed the post-survey or reported they read the book in the post-survey. Only participants who completed both surveys were included in the analysis that estimated the effect of book club experience on empathy scores. Since the project identifiers matched baseline to post-intervention surveys, changes in empathy scores were calculated per participant. We divided the matched sample into three categories based on their intervention status (i.e., did not read/listen to the book (Control A), did read/listen to the book but did not participate in a book club session (Control B), did read/listen to the book, and did participate in a book club session (Intervention). The Q-Q plot of difference was used to assess data distribution and found no severe violation of the normality assumption. Thus, a paired-sample t-test was used to assess the empathy score change in the Intervention group by comparing baseline and post-score, and one-way analysis of variance (ANOVA) was used to compare the empathy score change between the groups using difference scores. A p-value < 0.05 was considered statistically significant. Statistical analysis was performed with SAS 9.4 (SAS Institute Inc., Cary, NC, USA).

The qualitative component used an exploratory-descriptive approach, and thematic analysis was conducted using Braun and Clarke’s method [16]. Two members of the research team (KP, AJM) independently read and re-read the verbatim book club transcripts to immerse themselves in the data, making detailed notes to identify codes. Codes were then connected and collated into preliminary themes. The researchers then met to discuss their analyses, which were similar in focus leading to identification and agreement on final themes. Discussion and review of the themes also yielded key quotes to enrich understanding. This process was led by a member of the research team with qualitative research experience (KP), and an audit trail was recorded during the individual and group analyses. To increase the validity and reliability of the findings, themes were then reviewed and confirmed by a third researcher (FC) and the two psychologists (AMG, SP) who facilitated the book club sessions. The findings were not returned to book club participants for checking. Triangulation of data from the post-intervention survey’s open-ended questions and the book club discussions was performed.

## Results

Empathy score

Seventy-four (28.6%) participants completed the baseline survey and 73 (28.2%) completed the post-intervention survey. Table 1 summarizes responder numbers by role and experience. At baseline, there was no significant difference in TEQ empathy scores between role groups (F=1.40, p=0.25). Kruskal-Wallis tests found no association between baseline empathy scores and the number of years in practice for attendings (p=0.47), CRNAs (p=0.63), or residents (p=0.85).

**Table 1:** Survey Responder Demographics CRNA, Certified Registered Nurse Anesthetists; PGY, Postgraduate Year; CA, Clinical Anesthesia; PA, Physician Assistant; NP, Nurse Practitioner.

Role	Years’ Experience	Baseline	Post
Attending	<5	11 (14.9%)	11 (15.1%)
	5-10	8 (10.8%)	11 (15.1%)
	>10	17 (23.0%)	14 (19.2%)
CRNA	<5	3 (4.1%)	4 (5.5%)
	5-10	2 (2.7%)	1 (1.4%)
	>10	8 (10.8%)	8 (11.0%)
Resident	Intern (PGY1)	2 (2.7%)	5 (6.9%)
	CA1 (PGY2)	7 (9.5%)	3 (4.1%)
	CA2 (PGY3)	5 (6.8%)	5 (6.9%)
	CA3 (PGY4)	3 (4.1%)	2 (2.7%)
Other (PA, NP, etc.)		8 (10.8%)	9 (12.3%)
Total		74 (100.0%)	73 (100.0%)

Forty-two participants completed both baseline and post-intervention surveys. Among these participants, 14 did not read or listen to the book (empathy score: pre = 47.43±4.31, post = 48.14±4.29), seven reported reading or listening to the book but did not attend a book club session (empathy score: pre = 49.14±6.77, post = 49.29±4.46), and 21 attended a book club session in addition to reading/listening to the book (empathy score: pre = 47.90±5.03, post = 49.38±4.74). The paired-sample t-test showed that empathy scores trended upwards (Cohen’s d=.45, considered a small effect size) for the book club participants (n=21), but the change was not statistically significant (p=0.05, 95%CI of difference score (post-baseline) = (-.02, 2.97)). ANOVA did not find any significant difference in the average empathy score change between the three groups (F(2, 39) = 0.42, p=0.66).

Book club session themes

Twenty-five participants completed a book club session, with representation from all professions invited to participate (attendings, residents, CRNAs, advanced care providers). Four themes emerged during the thematic analysis of the book club sessions: 1) A Wake-up Call, 2) Deciding Whether to Take Action, 3) Learning and Nurturing Empathy, and 4) Changing the Culture. Review of open-ended responses from the post-intervention survey confirmed these themes and did not yield additional themes.

Theme One: A Wake-up Call

Throughout book club sessions, participants discussed empathy as an essential part of clinician practice. They voiced dismay over the author’s experiences with non-empathetic care and felt such experiences are unfortunately not uncommon: “it made me cringe because I know that happens a lot” (Attending_DB). Reading the book was described as “a wakeup call” (Attending_DB) to examine one’s practice and the overall culture. With their awareness stimulated about need for change, participants identified factors that influence empathetic practice and made recommendations for practice change.

Participants felt the practice of empathy is influenced by one’s experiences. Participants described how repeatedly observing others display a lack of empathy can subconsciously influence one’s behaviors, while conscious behavior changes can also occur as a means to fit in. The factor most discussed was becoming hardened to an extent that the human experience is forgotten: “The traumas you experience and don’t have a place to really talk about them…kind of hardens you…we become so far removed that we forget to have the human reaction” (Resident_CA). From a systems perspective, the major factor felt to impair empathetic practice is lack of time, causing clinicians to feel rushed during patient encounters which “can lead to a disconnect between the patient and team” (Resident_BA). Less commonly, participants described the negative effects of electronic charting (facing the computer, not the patient) and confidentiality regulations (depersonalization from not verbalizing patient names).

As participants reflected on these barriers and the author’s experiences, they discussed recommendations to help improve clinicians’ practice. One participant summarized empathy as “being one with the patient and being in the moment” (Attending_AB), yet participants felt clinicians can be “caught up in what we are medically and not as mindful as we should be” (Attending_DC). Although they work in fast-paced environments, spending time was deemed beneficial to both patients and clinicians: “When you are working these insane hours and stressful situations all day long…what else is there? Knowing how grateful that person is makes it worthwhile…you made a difference” (Resident_AA). The author’s experiences were described as “a good reminder to give our patients time to talk…and listen to them as well” (Attending_BA) and they recommended pausing prior to patient encounters to ready one’s self to “not just talk in the general direction of someone but actually talk WITH them” (Attending_CD). Participants also repeatedly discussed how the book made them more aware of how much patients hear and remember. They recommended to “choose your words carefully” (Attending_BA) to avoid insensitive communication, such as joking and labeling patients, that often occurs as a way to cope. The author’s experiences served as “an incredibly stark reminder there is a person behind here which we sometimes forget” (Attending_CB). To help prevent depersonalization, some imagined being in the patient’s situation; however, others cautioned that patients are the experts of their lived experience and “you shouldn’t always try to be in that place…because you’re not them ultimately” (Attending_DA). One explained: “We go into a patient encounter thinking, well-intentioned, of what we think is right. We allow that to guide the way we interact…which instantly takes autonomy away from patients” (Attending_CA). Instead, clinicians should get to know and respect what’s important to the patient.

Theme Two: Deciding Whether to Take Action

The discussions indicated that participants had increased awareness of the need and strategies for change, and they were asked if the book club would change their practice. Several discussed intended practice changes, while others shared how they had already adopted changes. For example, “the language I tried to use for the last week was totally different and my experience in the ICU was infinitely different than it had been prior to reading the book” (Resident_BB). Additionally, some discussed having advocated for empathetic care with colleagues: “I was hearing them say things and was like ‘our patients can probably hear us…just be more mindful.” I think everyone in medicine can really afford to hear that once in a while, as hard as it is” (PA_AA).

While many indicated they would work to change their practice, some felt their care was fully empathetic and did not require change. For these participants, it was felt that the book was “reaffirming” (Attending_AA). However, there were conflicting comments from some participants who felt they already practiced with a high level of empathy. Contradictory examples included a stated desire to be recognized by patients as the expert who knows best and questioning whether heightened empathy may be more important when the patient is a clinician as was the case of the author.

Theme Three: Learning and Nurturing Empathy

When asked if the book should be part of medical curricula, participants discussed how they had learned empathy, which revealed differences according to profession. Those with a nursing background discussed learning empathy by “spending hours with patients…being that person at the bedside” (CRNA_AB). Conversely, those with a medicine background indicated that they had not received formal training on empathetic care and felt current efforts to integrate it into medical education should continue to expand.

Experiential learning was deemed a powerful way to learn about the patient experience and improve practice of empathy. Several discussed how having been a patient “gives you a different perspective…it reminds you to be very careful about what you say because you never know how things are heard” (CRNA_AA). From an education perspective, using storytelling, role play, and simulation were identified as effective experiential techniques for teaching and nurturing empathy. Experiential learning in clinical settings was deemed particularly beneficial because “a lot of it comes with practice and experience…the more you do it, the more you are placed into situations…the better you will get” (Attending_BA). Participants also discussed the importance of leading by example when working with trainees in clinical settings. Giving feedback to trainees was felt to be an important strategy to help develop empathy, and the book prompted participants to change their feedback approach to better model empathy: “I’ve developed a way on the chart (to indicate) ‘we’ll talk about this later.’ Then go over it later, rather than in front of the patient. I used to do things in the moment, and I’ve been learning not to do that because sometimes the patient is awake” (Attending_DB).

Overall, participants supported using book club as a teaching tool. They felt the In Shock book was particularly powerful for teaching empathy. It was described as “a tangible way to put yourself in her shoes” (Attending_AA) and can “raise awareness because unless you had some of these experiences to draw from, you can’t understand what it feels like” (CRNA_AA). Participants felt reading the book encouraged reflection, but that its effect is maximized by “getting together and talking and learning from each other” (Resident_AA). There were various opinions about where empathy-focused book club would best fit within medical curricula. Some suggested early on in one’s training, while others felt it would be more impactful after gaining some experience. Revisiting it was also suggested to overcome hardening and decline in empathy: “Frequent reminders about humanizing stuff is really helpful…to get people to come back to where they meant or intended to be” (Attending_CC).

Theme Four: Changing the Culture

A major focus of the book was the need to improve clinician self-care because compassion toward yourself improves empathy towards others. One participant stated it is important “to restore humanity for the patient’s sake and also for our own sake” (Attending_CA). Participants discussed examples from the book in which clinicians were encouraged not to show emotions and confided this also happened during their training. The consequences were explained by a participant: “We pride ourselves on our M&M (Morbidity & Mortality rounds) conversations and how much we look for problems we can fix, but we never talk about: ‘How did that make you feel?’ That hardens you” (Resident_CA). Another voiced frustration: “Compassion...when did this become a weakness? This is something that really spoke to me” (Resident_AA). The book club was valued as an opportunity to discuss feelings and ways to improve the culture.

Participants stressed the need to normalize open discussion: “Maybe we could have a culture of just being open about how challenging our job is, how stressful it is, and the emotional toll it takes on us as human beings. Just let that be a natural thing so that when a trainee has that experience, it’s not a foreign thing” (Attending_DE). The need for a safe place to express emotions and someone to talk to was repeatedly discussed. However, some felt fears of punitive actions or being made fun of contributing to a culture of silence. It was suggested that those who work with trainees have a responsibility to “allow yourself and your team to stop and pause and reflect on the humanity of a difficult situation” (Attending_CA). One resident explained their desire for such support: “I think the level of support kind of wanes as we progress through training. We want to be more independent and have attendings be more hands off, but I don’t think that means we want less emotional support” (Resident_CA). The value of debriefing, especially after traumatic events, was emphasized as a helpful way to both facilitate learning and provide support. Although debriefing was desired, the varying schedules and fast pace of surgical environments caused participants to be unsure of how it could be operationalized.

## Discussion

We did not find any significant impact of participating in book club sessions on empathy score change. One result worth noticing is the baseline empathy score of our sample was high. The average empathy scores of undergraduate samples used in developing and validating the TEQ ranged from 44.54 to 47.27 [15]. The average baseline score for all matched participants in this study was 47.95, which suggests the baseline empathy level of our sample is comparable to, if not higher than, the reported population level. Thus, there might have been limited room for improvement in empathy scores following the intervention. This finding is not consistent with mainstream findings in the literature suggesting that empathy in clinicians and trainees is at risk. The baseline empathy score in our sample may be explained in a few ways. This was a self-selected group of participants who may have chosen to participate due to heightened interest in promoting clinical empathy. It is also possible that the trivial change in self-ratings of empathy can be convincingly attributed to medical training or practice, and there is not a serious decline in empathy during medical education and experience [7]. Other educational and wellness programs our institution had previously implemented to promote clinical empathy may also account for the high baseline scores. 

Thematic analysis of the book club sessions provided further insight into the sample’s empathy. Participants repeatedly discussed empathy as an essential part of clinician practice. Knowing this practice expectation and being aware of the study focus may have resulted in social desirability response bias and the high self-reported TEQ scores. On the other hand, Theme one described that, although participants were aware that practicing empathetic care is of high importance, there is a need for improvement in clinicians’ practice. The book club served as “a wakeup call” to participants and resulted in self-examination of their practice of empathy. Participants were able to identify barriers and draw upon the author’s experiences to make recommendations, such as avoiding insensitive communication and rushing patient encounters, to help themselves and other clinicians practice with heightened empathy.

The book club also led to intended and actual practice change among participants. This is consistent with our post-intervention survey results where majority of the sample indicated they would practice differently after reading the book. Theme two also described how some participants felt they already practiced with a high level of empathy and did not need to change their practice. This too was consistent with the post-intervention survey in which 20.6% of participants reported their practice would not change. Yet, there were conflicting comments such as a desire to be recognized as the one who knows best and questioning whether empathy is most important for educated clinician patients. This may indicate a potential mismatch between self-report of empathy and the actual practice of empathy. It is unclear if high empathy scores as measured by TEQ translate to empathetic patient care. The relationship between patient care and empathy scores may not be linear. It is assumed that clinicians who have low empathy scores will not relate well to patients and will have a difficult time providing compassionate care. However, it may also be that those with high empathy scores could struggle because they are too emotionally involved.

Discussions on learning empathy (theme three) were prevalent, in part due to question prompts. Book club discussions brought out that many participants felt they had a lack of formal training in empathy, but also emphasized that empathy is learned by emulating others and from personal experience. Participants felt that while empathy can be learned in a variety of ways, it is important to purposefully teach it throughout medical curricula. Furthermore, there is a need to revisit empathy throughout one’s career because of the effect that becoming “hardened” over time can have on one’s practice. Reading In Shock and participating in an accompanying book club was felt to help revisit empathy. The diverse composition of our book club promoted exchange of strategies learned through experience (attendings and CRNAs) and recent education (residents) and this approach is recommended to increase exposure to different views and methods for enhancing empathy in practice. Furthermore, theme four details how the book was particularly helpful in stimulating discussion about the need for culture change in medical training and work environments. The importance of practicing empathy toward self, trainees, and fellow clinicians was stressed. Yet, participants struggled with knowing how culture change could be actualized. Book clubs may provide a safe place for the open discussions yearned for by participants.

Our study is subject to several limitations. First, the results are based on a convenience sample from a single anesthesiology department in the US, which limits study power and generalizability. Second, only a small portion of the anesthesiology team responded to both surveys. This sample of clinicians may independently seek strategies, like this study, to nurture their empathy. Thus, self-selection bias, an issue that most survey studies are subject to, should be kept in mind when interpreting the results. Also, we determined the participants’ group status based on whether they read/listened to the book and whether they participated in a book club session, which is also subject to self-selection bias. Follow-up studies utilizing a randomized control design are warranted to address the self-selection bias. Third, our study is underpowered. A post hoc power analysis revealed a needed sample size of n=25 to detect a significant difference in means of 1.48 (i.e., baseline=47.9, post-intervention=49.38) in the Intervention group, assuming a standard deviation of differences of 3.3 and correlation of 0.78, using a paired t-test with a 5% two-sided significant level. Despite being underpowered, as a pilot study examining the effect of the book club as an empathy intervention, the quantitative results provide preliminary data for future studies. Fourth, we used the TEQ because it is widely utilized in medical education studies, and the items are aligned with the clinical empathy definition used in this study. However, questions have been raised regarding the validity of using self-report questionnaires to measure perceived empathy in practice, as it often fails to consider clinical and institutional contexts [11,17]. This may be one explanation for why the results are contradictory to the meta-analysis on rates of empathy in medical education. We tried to address this limitation with qualitative components to capture richer data and add context to the findings. Additionally, the survey was anonymous to address potential response bias, but participants could have rated themselves as more empathetic on surveys because empathy is desired in clinicians.

## Conclusions

To conclude, our survey results do not provide evidence to support the notion that participation in a book club significantly increases empathy scores as measured by the TEQ. Qualitative analysis results suggested that book clubs may be a viable venue for initiating important discussions on empathetic practice. Book clubs are a long-standing but underexamined educational strategy for providing a safe place to debrief, share experiences, express emotions, and normalize compassion. However, just one book club session does not seem to result in any substantial changes in clinical empathy scores. Further examination of this low-cost educational strategy is warranted.
